# Post-traumatic stress disorder and self-reported outcomes after traumatic brain injury in victims of assault

**DOI:** 10.1371/journal.pone.0211684

**Published:** 2019-02-07

**Authors:** Dominic Bown, Antonio Belli, Kasim Qureshi, David Davies, Emma Toman, Rachel Upthegrove

**Affiliations:** 1 College of Medical and Dental Sciences, University of Birmingham, Birmingham, United Kingdom; 2 Institute of Inflammation and Ageing, University of Birmingham, Birmingham, United Kingdom; 3 National Institute for Health Research, Surgical Reconstruction and Microbiology Research Centre, Birmingham, United Kingdom; 4 University of Central Lancashire, Preston, United Kingdom; 5 Institute for Mental Health, University of Birmingham, Birmingham, United Kingdom; Stellenbosch University, SOUTH AFRICA

## Abstract

**Introduction:**

Assault is the third most common cause of traumatic brain injury (TBI), after falls and road traffic collisions. TBI can lead to multiple long-term physical, cognitive and emotional sequelae, including post-traumatic stress disorder (PTSD). Intentional violence may further compound the psychological trauma of the event, in a way that conventional outcome measures, like the Glasgow Outcome Scale (GOS), fail to capture. This study aims to examine the influence of assault on self-reported outcomes, including quality of life and symptoms of PTSD.

**Methods:**

Questionnaire were completed by 256 patients attending a TBI clinic, including Quality of Life after Brain Injury (QOLIBRI) and PTSD checklist (PCL-C). Medical records provided demographics, clinical data and aetiology of injury. Subjective outcomes were compared between assault and other causes.

**Results:**

Of 202 patients analysed, 21% sustained TBI from assault. There was no difference in severity of injuries between assault and non-assault groups. No relationship was found between self-reported outcomes and TBI severity or GOS. The assault group scored worse in all self-reported questionnaires, with statistically significant differences for measures of PTSD and post-concussion symptoms. However, using threshold scores, the prevalence of PTSD in assaulted patients was not higher than non-assault. After adjusting for age, ethnicity and the presence of extra-cranial trauma, assault did not have a significant effect on questionnaire scores. Exploratory analysis showed that assault and road traffic accidents were associated with significantly worse outcomes compared to falls.

**Conclusion:**

Quality of life is significantly related to functional and psychological outcomes after TBI. Assaulted patients suffer from worse self-reported outcomes than other patients, but these differences were insignificant when adjusted for demographic factors. Intentionality behind the traumatic event is likely more important than cause alone. Differences in quality of life and other self-reported outcomes are not reflected by the Glasgow Outcome Scale. This information is useful in arranging earlier and targeted review and support.

## Introduction

Traumatic brain injury (TBI) is a traumatically induced disruption of brain function, manifesting as loss of consciousness or memory, alteration in mental and/or focal neurological deficits. TBI is a leading cause of death and disability in both developing and developed countries [1]. The incidence of TBI varies between 150–300 cases per 100,000 population per year [1], and is associated with younger age and male gender [2]. The three most common causes of TBI are falls, road traffic collisions (RTC) and assault, with assaults accounting for around 18% of all cases in western populations, and around 40% of cases for younger patients [2]. TBI due to assault is also strongly associated with being male, substance misuse, low income, and minority ethnicity [2–4]. While TBI mortality has reduced by 30–40% in recent years [5], more victims are surviving with multiple chronic sequelae. Patients may develop post-concussion syndrome (PCS), characterised by physical symptoms, including headaches; cognitive problems, such as difficulty concentrating; and emotional issues, like irritability [6].

TBI is the third largest contributor to injury-related hospital costs in Europe [7], and accounts for the highest number of years lived with disability [8]. This disability restricts patients’ livelihoods, negatively affecting their self-image, coping mechanisms and health-related quality of life (HRQoL) [9]. While patients’ functioning improves over time, evidence suggests most improvement occurs in physical domains, while communication-related, cognitive and emotional problems remained more constant over time [10]. HRQoL also remains adversely affected in the long term, with studies finding significant reductions 2, 5, and 10 years post injury [10,11].

Interpersonal violence carries a highly significant emotive context that can compound the burden of TBI. Previous research has shown that survivors of TBI from assault suffer from worse symptoms, are more significantly disabled, less productive, a larger burden on caregivers, show less community reintegration, and gain less from inpatient rehabilitation [12–14]. The literature is mixed, however, with some studies finding no significant influence of mechanism of injury on functional and cognitive outcomes [15,16]. Few of these studies regarding assault in TBI focus on HRQoL, mostly examining clinician-rated outcomes, which will not necessarily capture the subjective experience of long term sequelae [12–16].

The psychological consequences of TBI are significant, doubling the risk of developing subsequent psychiatric illness [17], in particular, anxiety, depression, and post-traumatic stress disorder (PTSD). PTSD is characterised by intrusion and re-experiencing of memories of a traumatic event, avoidance of memory triggers, emotional numbing, and hypervigilance. Research suggests that 17–33% of people develop PTSD after TBI [18,19]. The relationship is complex, however, with some evidence that patients with severe TBI have a reduced risk [19]. Importantly, two studies of soldiers with mild TBI found that the relationship between TBI diagnosis and poor outcomes became insignificant when adjusted for PTSD and depression [20,21], emphasising the importance of psychiatric comorbidities in patients’ recovery. Evidence consistently shows that violence is more often associated with PTSD than other forms of trauma [22], with both criminal assault and intimate partner violence being major risk factors [23,24]. The evidence is mixed, however, as to whether the risk of PTSD is compounded in assault victims who suffered also from TBI [14,16].

TBI is generally classified by its severity, according to Glasgow Coma Scale (GCS) score: the National Institute of Health and Care Excellence (NICE) classifies a score of 13–15 as mild TBI, 9–12 as moderate, and ≤8 as severe [25]. CT scans of the head can be graded according to the Marshall classification system, producing six categories of severity. This is primarily concerned with the presence of swelling, as determined by midline shift and/or compression of basal cisterns, and the presence and size of contusions and haemorrhages [26].

Most research that differentiates between aetiology of TBI focuses on socio-demographic characteristics or objective functional outcomes. This project hypothesises that rates of PTSD would be higher in patients who had suffered from assault than those who had an unintentional cause of TBI, and that this increased rate of PTSD could potentially account for some of the worse outcomes experienced by these patients. Better understanding of the influence interpersonal violence has on outcomes after TBI could lead to more targeted rehabilitation strategies, improving long-term recovery.

## Aims

This study aims to examine the influence of assault, compared to other aetiologies, on HRQoL, symptoms of post-traumatic stress and other subjective outcomes in victims of TBI.

## Methods

This is a cross-sectional study of 256 TBI patients attending the multidisciplinary TBI clinic for follow-up review at University Hospital Birmingham NHS Foundation Trust (UHBFT). The study consists of secondary analysis of an anonymised database of all patients who attended between August 2013 and February 2016. UHBFT is a major level-1 trauma centre that provides adult neurotrauma services to the Birmingham urban area and surrounding rural counties, with a catchment population of approximately 4 million. Patients are referred to the TBI clinic from hospitals within the catchment area.

As part of routine care, patients who attended the clinic completed the following assessments:

Quality of Life after Brain Injury Questionnaire (QOLIBRI) [27]: a questionnaire developed specifically to subjectively assess HRQoL and cognitive function in victims of TBI. It consists of 37 items in two parts: Part A assesses satisfaction levels and comprises four categories: Cognition (7 items), Self-perception (7 items), Autonomy in daily life (7 items), and Social relationships (6 items). Part B assesses the burden of symptoms and is composed of two categories: Negative emotions (5 items) and Physical problems (5 items). Patients’ responses are 5-point Likert-type: “Not at all”, “Slightly”, “Moderately”, “Quite”, or “Very”. The responses are quantified from 0–4, with burden responses (part B) reversed. The overall score is obtained by averaging the score of the answered questions and multiplying by 25. If at least two thirds of questions are answered, scores remain reliable so were included in analysis. Scores range from 0 to 100.

Post-traumatic Stress Disorder checklist (civilian version) (PCL-C) [28]: a 17-item questionnaire that assesses PTSD symptoms in the previous month. Questions are anchored to “stressful experiences from the past”. Questions can be grouped according to symptoms of intrusion and re-experiencing (5 items), avoidance (2 items), numbing (5 items), and hyperarousal (5 items). Responses are categorised using a Likert scale, quantified from 1–5 and scored by summation, with a maximum of 85. The checklist can be used for diagnosis using a threshold of 36, 44, or 50, depending on expected population PTSD prevalence. Individual items can be scored by treating responses of 3–5 as symptomatic and 1–2 as asymptomatic.

Impact of Event Scale (IES) [29]: a 15-question measure of symptoms of post-traumatic stress, including intrusive symptoms, avoidance, and emotional numbing, about a particular event. It does not measure hyperarousal so is not valid for diagnosis of PTSD. Questions refer to how often a particular feeling or thought process occurred in the previous week, and responses are “not at all”, “rarely”, “sometimes”, or “often”, and are scored 0, 1, 3, and 5 respectively. The maximum score is 75, obtained through summation.

Patient Health Questionnaire (PHQ-9) [30]: a 9-item screening tool for depression that assesses how much symptoms of depression have impacted patients in the last two weeks. Patients respond to each item, referring to a different symptom, with “not at all”, “several days”, “more than half the days”, or “nearly every day”. Responses are quantified from 0–3 and summed. Meta-analysis has found that the PHQ-9 has acceptable diagnostic properties for major depressive disorder using a cut-off score between 8–11, out of a total of 27.

Rivermead Post-concussion Questionnaire (RPQ) [31]: this measures the severity of 16 symptoms of post-concussion syndrome over the previous 24 hours, compared with before head injury. Symptoms are rated from 0–4, with 0 representing “not experienced at all” and 4 representing “a severe problem”, and with total scores from 0–64. The RPQ contains two empirically distinct clusters, the RPQ-3 and RPQ-13 subscales: the first three items (headaches, dizziness, nausea and/or vomiting) represent “early” symptoms, whilst the other 13 items represent “late” symptoms. These subscales will be analysed in addition to overall score.

Headache Impact Test (HIT) [32]: a 6-part test of the impact of a patient’s headaches on social and cognitive functioning, vitality, and psychological distress. It also measures severity of headaches. Questions related to the past 4 weeks, and answers are rated on a 1–5 scale of “never” to “always”. Scores are obtained through summation, ranging from 6–30.

All patients completed the QOLIBRI, RPQ and either the PHQ or PHQ-9. 35 patients did not complete the PCL-C, IES and HIT, as these were introduced into the survey battery at a later date. A higher score indicates worse outcomes for all questionnaires but the QOLIBRI, where lower scores indicate worse outcomes.

Patients personally completed questionnaires while attending the clinic, with a clinical team member in attendance to help with methodological or vocabulary difficulties, but not influencing responses. Patients were allowed to discuss questions with their carers but responses were only taken from the former.

The responses of patients who returned questionnaires were compiled in a database. Medical records of these patients were reviewed retrospectively to collect demographic and clinical data, including:

- Mechanism of injury
- TBI severity, according to Glasgow coma scale (GCS) on admission: severe (GCS score 3–8), moderate (9–12) and mild (13–15).
- Cranial CT scan, according to the Marshall classification
- Glasgow outcome scale (GOS) score on discharge
- Hospital management (conservative or neurosurgical intervention)
- Number of days hospitalised
- Number of days in an intensive care unit (ITU)
- Extracranial trauma or pre-existing medical conditions
- At-scene loss of consciousness
- Post-traumatic amnesia
- Involvement of alcohol during event
- Litigation activities

If a patient attended the clinic on multiple occasions, only questionnaires from their first visit were analysed. Patients were excluded from the analysis if the cause of their injury could not be ascertained; if the injury was caused in combat (to exclude the potential confounder of military occupation or environment) or if patient had not suffered a traumatic brain injury. Incomplete questionnaires were excluded from analysis if patients did not respond to one or more questions, aside from the QOLIBRI, where scores are calculated from averages and remain reliable if two thirds of questions are answered.

Ethical approval for this study was obtained from the NHS Health Research Authority–reference 17/LO/0153.

### Statistical analysis

Demographics, clinical data and subjective measures were compared between assaulted and non-assaulted TBI patients. Continuous variables were compared using the Mann-Whitney U Test and categorical variables using the chi-square test. Comparisons of questionnaire scores amongst severity, CT Marshall grades, and GOS score were performed using the Kruskal-Wallis test. Correlations between numerical variables were assessed using Spearman’s rank correlation coefficient. Multiple linear regression analysis was performed for each questionnaire, with cause of injury as the independent variable. Demographics that either correlated with or had an effect on each questionnaire were also included as independent variables. With all tests, pairwise was used for missing data or incomplete questionnaires to ensure maximal usage of data. A nominal significance level of p = 0.05 was used, and Bonferroni correction was used for multiple testing of items in individual questionnaires. Analysis was undertaken using the Statistical Package for the Social Sciences version 22.0 software.

## Results

### Sample demographics

Of 256 patients on the database, 202 were included in analysis. Six were excluded as they did not suffer from TBI, four for injuries caused in combat and 44 for unknown aetiology of injury. 21% (n = 43) of included patients sustained their injury due to assault, with the remainder (non-assault group) sustaining injury from falls (40%, n = 81), road traffic collisions (33%, n = 66), and other causes (6%, n = 12). The median time from injury to follow-up was 5.1 months (inter-quartile range (IQR) 3.6–7.7)).

Comparisons of demographics and clinical characteristics are presented in Table 1. Assaulted patients tended to be younger and non-Caucasian, and required shorter stays in both intensive care and general hospital wards when compared with the non-assault group. Assaulted patients had a lower incidence of extracranial trauma and pre-existing conditions, but were more likely to report the involvement of alcohol or loss of consciousness during the traumatic event.

**Table 1:** Comparison of demographics and clinical characteristics between assaulted and non-assaulted patients.

Demographics		Mechanism of TBI		Number valid (%)	p-value
		Non-assault	Assault		
Number of patients		159	43	202 (100)	
Median Age (IQR)		50.4 (26.5–63.4)	29.3 (22.1–36.4)	179 (88.6)	**<0.001**
Proportion Male		74.2%	90.7%	202 (100)	**0.023**
Ethnicity	White	87.9%	72.1%	200 (99.0)	**0.014**
	Asian	7.6%	23.3%		
	Other	4.5%	4.7%		
Median follow-up interval (IQR) [months]		5.1 (3.6–7.6)	6.2 (3.7–8.3)	174 (86.1)	0.606
Severity (GCS)	Severe	21.8%	14.0%	190 (94.1)	0.398
	Moderate	10.9%	16.3%		
	Mild	67.3%	69.8%		
Median Marshall Classification (IQR)		2 (2–4)	2 (2–3)	170 (84.2)	0.908
Glasgow Outcome Score	3	21.8%	0.0%	105 (52)	0.122
	4	25.7%	37.0%		
	5	52.6%	63.0%		
Median days hospitalised (IQR)		11 (5–22)	5 (3.25–14)	175 (86.6)	**0.015**
Median days ITU (IQR)		0 (0–6)	0 (0–1.75)	176 (87.1)	**0.047**
Extracranial trauma from event		36.5%	20.9%	202 (100)	0.068
Previous co-morbidities		50.3%	25.6%	202 (100)	**0.005**
Neurosurgery required		32.0%	36.6%	191 (95.6)	0.579
Loss of Consciousness at scene		55.9%	75.8%	151 (74.8)	**0.045**
Post-traumatic amnesia		25.2%	30.2%	202 (100)	0.559
Alcohol involved during injury		33.3%	56.3%	134 (66.3)	**0.024**
Litigation after injury		4.4%	4.7%	202 (100)	0.944

**]**All patients completed the QOLIBRI questionnaire sufficiently to be included in analysis (n = 202). Fewer patients completed the RPQ (n = 177), PHQ-9 (n = 160), PCL-C (n = 144), HIT (n = 148), and IES (n = 129). For the PCL-C, HIT and IES, 35 fewer patients were offered the questionnaire. All other variation is due to incomplete responses being excluded from analysis.

### Severity of TBI

The severity of TBI ranged from mild to severe based on GCS on admission (mild: 67.9%, moderate: 12.1%, severe: 20.0%). Although the injuries tended to be more severe in the non-assault group, according to both GCS and Marshall classification, and assaulted patients tended to have better outcomes on discharge according to GOS score, these differences were not significant. Additionally, no significant effect of severity was found on any of the questionnaire scores, nor did questionnaire scores vary significantly between GOS scores. However, CT Marshall grade did have an effect on PCL-C score (p = 0.031), with more severe Marshall grades associated with more favourable PCL-C scores.

### Subjective measures

All questionnaires showed a possible trend towards worse outcomes for patients in the assault group(see Table 2). Only the differences in IES scores were significant (Non-assault versus assault respectively; IES: 9 (0–34) vs 30 (8–44), p = 0.032). Differences between RPQ (full) and PCL-C scores were of borderline significance (RPQ: 19.0 (7.0–37.5) vs 31.0 (14.3–42.0), p = 0.057; PCL-C: 23.5 (18.0–46.8) vs 35.0 (22.5–48.8), p = 0.073). Assaulted patients scored significantly worse on RPQ-3 subsection, (RPQ-3: 3 (0–6) vs 5 (2–6.3), p = 0.020), but no QOLIBRI domain showed any significant change.

**Table 2:** Comparison of self-reported outcomes between assaulted and non-assaulted patients.

Questionnaire (Median score +IQR)	Number of valid responses	Mechanism of TBI		p-value
		Non-assault	Assault	
**PCL-C**	144	23.5 (18.0–46.8)	35.0 (22.5–48.8)	0.073
**QOLIBRI**	202	64.9 (52.1–82.6)	61.8 (51.4–77.7)	0.401
**RPQ (Full)**	177	19.0 (7.0–37.5)	31.0 (14.3–42.0)	0.057
**RPQ-3**	192	3.0 (0.0–6.0)	5.0 (2.0–6.3)	**0.020**
**RPQ-13**	182	16.0 (6.8–32.0)	25.0 (11.3–37.0)	0.098
**PHQ-9**	160	6.0 (1.0–16.8)	10.0 (2.3–15.8)	0.747
**HIT**	148	4.0 (0.0–12.0)	5.0 (1.0–15.0)	0.287
**IES**	129	9.0 (0.0–34.0)	30.0 (8.0–44.0)	**0.032**

When examining individual items in the PCL-C, more assaulted patients were symptomatic (score of 3–5) for “trouble falling or staying asleep” (p = 0.028) and “being ‘super alert’ or watchful on guard” (p = 0.001). Bonferroni correction gives a modified critical p value of p = 0.0029, so the latter result can be considered statistically significant. Diagnostic threshold scores of 36, 44 and 50 for the full checklist all showed higher prevalence of PTSD among assaulted patients, but none of these differences were significant (p = 0.213, p = 0.392, p = 0.810 respectively) (see Table 3).

**Table 3:** Comparison of prevalence of PTSD between assaulted and non-assaulted patients, according to PCL-C diagnostic thresholds.

PCL-C Threshold score	Proportion above threshold		p-value
	Non-assault	Assault	
**36**	33.6%	46.9%	0.213
**44**	29.2%	37.5%	0.392
**50**	20.1%	21.9%	0.810

### Univariate analysis

All six questionnaires correlated significantly with each other (see Table 4). Age also correlated significantly with each self-reported measure, with younger patients reporting worse outcomes, independent of the aetiology of their injury. Ethnicity had a significant impact, with Asian patients reporting significantly worse outcomes for RPQ, PHQ-9 and HIT. Patients suffering from extracranial trauma also reported significantly worse outcomes for QOLIBRI, RPQ, PCL-C and PHQ-9. Neither time in hospital nor in ITU correlated significantly with any measure, and questionnaire scores did not vary according to sex, patients’ pre-existing co-morbidities, receiving neurosurgery, or loss of consciousness or alcohol involvement.

**Table 4:** Correlation between questionnaire scores and continuous demographic and clinical data.

	PCL-C	QOLIBRI	RPQ	PHQ-9	HIT	IES
**PCL-C**		r = -0.751 p < 0.001	r = 0.853 p < 0.001	r = 0.863 p < 0.001	r = 0.662 p < 0.001	r = 0.746 p < 0.001
**QOLIBRI**	r = -0.751 p < 0.001		r = -0.787 p < 0.001	r = -0.787 p < 0.001	r = -0.592 p < 0.001	r = -0.559 p < 0.001
**RPQ**	r = 0.853 p < 0.001	r = -0.787 p < 0.001		r = 0.875 p < 0.001	r = 0.764 p < 0.001	r = 0.706 p < 0.001
**PHQ-9**	r = 0.863 p < 0.001	r = -0.787 p < 0.001	r = 0.875 p < 0.001		r = 0.658 p < 0.001	r = 0.606 p < 0.001
**HIT**	r = 0.662 p < 0.001	r = -0.592 p < 0.001	r = 0.764 p < 0.001	r = 0.658 p < 0.001		r = 0.550 p < 0.001
**IES**	r = 0.746 p < 0.001	r = -0.559 p < 0.001	r = 0.706 p < 0.001	r = 0.606 p < 0.001	r = 0.550 p < 0.001	
**Age**	r = -0.262 p = 0.003	r = 0.218 p = 0.003	r = -0.231 p = 0.003	r = -0.282 p = 0.003	r = -0.422 p = 0.001	r = -0.191 p = 0.003
**Time in Hospital**	r = 0.018 p = 0.843	r = -0.068 p = 0.373	r = 0.059 p = 0.469	r = 0.074 p = 0.395	r = -0.061 p = 0.502	r = -0.030 p = 0.753
**Time in ITU**	r = 0.120 p = 0.187	r = -0.086 p = 0.258	r = 0.105 p = 0.193	r = 0.133 p = 0.125	r = 0.035 p = 0.702	r = 0.000 p = 0.997

### Regression analysis

Regression analysis of each self-reported outcome is presented in Table 5. Whether patients were assaulted, age, ethnicity, and the incidence of extracranial trauma were included as independent variables, as these were significantly different (or borderline) between assault and non-assault groups, and also influenced self-reported outcomes. The coefficient of assault denotes the absolute increase or decrease in questionnaire score attributable to being assaulted. This is also presented as a percentage change relative to the range of scores available for each measure (the difference between maximum and minimum scores). With the exception of QOLIBRI, a positive change in questionnaire score indicates less favourable outcomes.

**Table 5:** The effect of assault versus other causes on self-reported outcomes, when adjusted for confounding factors.

Questionnaire	Unstandardised Coefficients of Assault		Questionnaire Score Range	% Change from assault	p-value
	B	Standard Error			
**PCL-C**	5.200	3.925	68	7.65%	0.188
**QOLIBRI**	-1.254	3.292	100	-1.25%	0.704
**RPQ (Full)**	5.708	3.495	64	8.92%	0.105
**RPQ-3**	0.575	0.608	12	4.79%	0.345
**RPQ-13**	4.462	2.940	52	8.58%	0.131
**PHQ-9**	-0.703	1.768	27	-2.60%	0.691
**HIT**	-0.540	1.611	24	-2.25%	0.738
**IES**	6.550	4.736	75	8.73%	0.170

After adjusting for confounders (age, ethnicity and extracranial trauma), assault appeared to have the greatest impact on the PCL-C, IES, and RPQ full and RPQ-13 subsection scores, but none of these changes were statistically significant (PCL-C: 7.95%, p = 0.188; IES: 8.73%, p = 0.170; RPQ: 8.92%, p = 0.105; RPQ-13: 8.58%, p = 0.131). Furthermore, while assaulted patients had worse PHQ-9 and HIT scores, adjusting for confounders nullified this effect.

### Post-hoc analysis

A post-hoc analysis of observed power was performed for the comparison on our primary outcome measure (QOLIBRI) between assault and non-assault groups. This revealed a sample size of n = 166 would have 95% power to predict a difference of 15 points on the QOLIBRI between assault and non-assault groups. This difference is likely the minimum change required to be clinically significant: previous studies have suggested that changes of at least 30% are required for the change to be meaningful [33]. Our sample size was in excess of this.

An exploratory analysis was performed to identify whether, if analysed separately, assault and road traffic collisions were significantly more likely to result in poor outcomes than falls or other causes of TBI. The three groups were compared on each measure using the Kruskal Wallis test, then each group was compared separately using the Mann-Whitney U test. The comparison of assault and RTC group is not shown as all p values were greater than 0.05. The results of the other comparisons are presented in Table 6.

**Table 6:** Comparison of self-reported outcomes between patients following assault, road traffic collision and fall/other causes.

Questionnaire score (Median +IQR)	Mechanism of TBI			p-values		
	Assault	RTC	Fall/Other	Kruskal-Wallis	Assault vs falls	RTC vs falls
**PCL-C**	35.0 (22.5–48.8)	42.0 (20.8–58.0)	20.0 (17.0–29.5)	0.000	0.003	0.000
**QOLIBRI**	61.8 (51.4–77.7)	58.5 (49.8–74.5)	68.2 (53.5–87.5)	0.028	0.092	0.011
**RPQ (Full)**	31.0 (14.3–42.0)	26.5 (13.5–40.0)	16.0 (4.3–33.8)	0.011	0.010	0.017
**RPQ-3**	5.0 (2.0–6.3)	4.0 (1.0–6.0)	2.0 (0.0–5.0)	0.009	0.003	0.046
**RPQ-13**	25.0 (11.3–37.0)	25.0 (12.5–33.4)	13.0 (3.3–30.0)	0.023	0.021	0.027
**PHQ-9**	10.0 (2.3–15.8)	14.0 (1.0–18.0)	4.0 (0.0–13.0)	0.014	0.146	0.005
**HIT**	5.0 (1.0–15.0)	8.0 (2.0–16.0)	2.0 (0.0–10.0)	0.021	0.062	0.009
**IES**	30.0 (8.0–44.0)	19.0 (5.0–38.0)	6.0 (0.0–24.5)	0.001	0.003	0.002

## Discussion

In this cohort, patients who suffered from TBI following assault were younger and less likely to be Caucasian, as expected from previous literature. Assaulted patients showed a possible trend towards worse subjective outcomes in all six questionnaires, with a significant difference in the IES, measuring symptoms of post-traumatic stress, and the first subsection of the RPQ, measuring early symptoms of post-concussion. However, there did not appear to be a significantly greater prevalence of PTSD in the assault group. Self-reported outcomes were independent of TBI severity (GCS) and of objective outcome scores (GOS). Younger patients and non-Caucasian patients scored less favourably, confounding results.

### Factors leading to worse health-related quality of life after TBI

Previous research suggests HRQoL after TBI is negatively impacted by the severity of post-traumatic stress, headaches, depression and extracranial trauma [34, 35]. This supports our results, as QOLIBRI score correlated significantly with each subjective outcome measure. Assaulted patients reported worse symptoms of post-traumatic stress and of post-concussion–particularly the early symptoms of headaches, nausea and dizziness. This did not, however, correspond with significantly worse QOLIBRI scores. It does not appear that being assaulted was an independent factor in HRQoL after TBI in our patients; indeed, no significant difference was observed and any effect would likely be confounded by an indirect effect of increased symptomatic burden.

There is significant debate regarding the extent to which the functional outcomes measured by the Glasgow Outcome Scale influence HRQoL: some studies suggest a positive correlation and some suggest no relationship [35–37]. The GOS gives disproportionate weight to physical deficits, being less sensitive to changes in cognitive and emotional areas. It can also suffer from ceiling effects, unable to detect significant differences among patients classed as “good recovery” [38]; this is important given evidence that, over time, physical components of HRQoL improve to a greater extent than mental components [10]. In our cohort, GOS did not correlate with QOLIBRI score, or any subjective measure, but this may lack sensitivity, as few of the non-assault and none of the assault group were classed in group 3, or “severe disability”, and GOS score was only recorded in 52% of patients in our study.

There is an unclear relationship between severity of TBI and HRQoL. Although evidence suggests that subjects with more severe injuries suffer worse cognitive and social functioning [38,39], this does not necessarily reflect satisfaction with daily functioning. Like ours, some studies found no connection between initial post-injury GCS and HRQoL [35], but others have found an inverse relationship: two studies demonstrated that, according to GCS, mild TBI patients experienced worse HRQoL than severe TBI [40,41], while increased coma length has also been shown to predict better long-term HRQoL [42]. This fits with our finding that patients with a severe Marshall classification reported more symptoms of PTSD: this effect could be explained by more severe injuries resulting in more peri-trauma amnesia, which has a protective effect against PTSD [43]. Overall, previous research supports our findings that subjective outcomes in TBI patients are not strictly determined by injury severity.

Previous literature has established that younger age is protective against adverse physical outcomes after TBI [44]. Furthermore, while most patients experienced mild cognitive decline following TBI, age appears to protect against this [45]. Other studies have reported negative or no correlation between age and HRQoL [36,40], although one study found adolescents suffered more emotional and behavioural problems than adult TBI victims [35]. It is surprising that age correlated so significantly with each of our self-reported measures, with younger patients reporting less favourable outcomes, both overall and when analysing assault and non-assault groups separately. In our cohort, it is possible that younger patients with better outcomes were less likely to attend follow-up than older patients.

### The influence of assault on post-traumatic stress

Assault is an intentional form of injury that is particularly intrusive to the victim. This study found that assaulted TBI patients reported higher IES and PCL-C scores than the non-assault group, but only the former difference was statistically significant. When considering individual components of the PCL-C, assault victims were more likely to be ‘symptomatic’ for two items measuring hyperarousal. Despite the evidence that assault leads to increased symptoms of post-traumatic stress, this did not translate to an increased prevalence of PTSD in assaulted patients, when considering PCL-C score thresholds. Furthermore, it is suggested that a change of 10–20 points in the PCL-C is required to be clinically significant [28]; regression analysis determined that being assaulted was only responsible for 5.2-point change, and this value was not statistically significant. It is therefore unlikely that the increased burden of PTSD symptoms following assault in TBI is clinically significant.

### Post-hoc analysis

Exploratory analysis comparing assault and RTC separately against falls and other causes of TBI showed that assault and RTC were more likely to cause significantly worse outcomes in nearly every measure, while there was no significant difference between assault and RTC outcomes. There is increasing evidence that the feelings of anger or injustice are associated with worse outcomes following trauma [46]; this would fit with our findings, with patients being more likely to suffer feelings of anger or injustice following assault or RTC as compared to falls.

### Limitations

The cross-sectional, retrospective nature of this study limits the confidence with which any correlations can be drawn from the data. Additionally, the study’s reliance on questionnaires for self-reported outcomes creates a possibility for response bias, while using the PCL-C to diagnose PTSD is not as reliable as the gold standard–a clinically structured interview. Notably, the differential diagnosis of PTSD from post-TBI symptoms is particularly difficult: both conditions can cause emotional numbing and social withdrawal, as well as comorbid conditions such as depression and generalised anxiety [47]. The IES and PCL-C, measuring symptoms of PTSD, and the RPQ, measuring post-TBI symptoms, all have questions regarding irritability, poor concentration, and sleep disturbance, so it is unsurprising that there was significant correlation between all three. Despite this overlap, the PCL-C is a recommended tool to screen for PTSD in TBI research [48]. The study could, however, have been improved by screening for symptom and diagnosis validity. Additionally, patients were separated according to whether they were assaulted or not, as a proxy for whether the injury was caused intentionally or by another party. This would not, for example, distinguish between a car accident that occurred as a fault of the injured party versus a hit-and-run scenario; it could be more useful to determine patients’ attributions of “intentionality”.

Information regarding potential mediators of observed relationships was highly reliant on data collected from patients’ clinical notes, which is not ideal: for example, length of coma, and socio-economic status can both influence recovery after TBI, but were not recorded in patients’ notes consistently enough to be analysed. Furthermore, many patients did not complete all questionnaires, reducing power of analysis, while the relatively small sample size and high p-values made regression analysis difficult to interpret. The study predominantly includes cases of mild TBI, and observed differences could be greater when considering moderate or severe TBI alone.

### Implications

These results suggest that following TBI, subjective outcomes are psychologically mediated, rather than due to organic factors like severity of injury. HRQoL correlates with symptoms of post-concussion syndrome, depression, and PTSD. There are significant potential benefits to rapidly identifying patients likely to suffer from persisting symptoms. The psychological trauma of TBI is significant and the relationship between brain injuries and the development of PTSD is complex, so all victims of TBI should be evaluated for potential adverse mental health outcomes.

Future research should investigate correlation between subjective outcomes and age, potentially examining differences between patients who suffer from TBI in a larger sample, recruited from discharge rather than follow-up. Development of measures of “intentionality” attribution could help to elucidate differences seen between victims of violence and other patients, and could explain why patients undergoing litigation tend to have worse outcomes. Such information would be invaluable to inform the targeting of strategies to treat adverse outcomes from TBI.

## Conclusion

Self-reported outcomes appear independent of TBI severity, and are more sensitive to patients’ needs than objective outcome scores. Assessment of cognitive and psychological function at follow-up is as important as assessing physical function, in order to ensure improvements to HRQoL. Assaulted patients were at a higher risk of developing symptoms of post-concussion and PTSD than all other patients, but this may not correspond with a higher prevalence of PTSD. Assault and RTC were associated with worse outcomes than falls or other causes.
